# There are four dynamically and functionally distinct populations of E-cadherin in cell junctions

**DOI:** 10.1242/bio.014159

**Published:** 2015-10-15

**Authors:** Zahra Erami, Paul Timpson, Wu Yao, Ronen Zaidel-Bar, Kurt I. Anderson

**Affiliations:** 1 Cancer Research UK Beatson Institute, Glasgow G11 7DU, UK; 2 Mechanobiology Institute, National University of Singapore, Singapore 117411

**Keywords:** E-cadherin, FRAP, Cell adhesion, Super-resolution microscopy

## Abstract

E-cadherin is a trans-membrane tumor suppressor responsible for epithelial cell adhesion. E-cadherin forms adhesive clusters through combined extra-cellular cis- and trans-interactions and intracellular interaction with the actin cytoskeleton. Here we identify four populations of E-cadherin within cell junctions based on the molecular interactions which determine their mobility and adhesive properties. Adhesive and non-adhesive populations of E-cadherin each consist of mobile and immobile fractions. Up to half of the E-cadherin immobilized in cell junctions is non-adhesive. Incorporation of E-cadherin into functional adhesions require all three adhesive interactions, with deletion of any one resulting in loss of effective cell-cell adhesion. Interestingly, the only interaction which could independently slow the diffusion of E-cadherin was the tail-mediated intra-cellular interaction. The adhesive and non-adhesive mobile fractions of E-cadherin can be distinguished by their sensitivity to chemical cross-linking with adhesive clusters. Our data define the size, mobility, and adhesive properties of four distinct populations of E-cadherin within cell junctions, and support association with the actin cytoskeleton as the first step in adhesion formation.

## Abstract (precis)

**Summary:** Four populations of E-cadherin are identified within cell junctions based on the molecular interactions which determine their size, mobility, and adhesive properties, and support association with the actin cytoskeleton as the first step in adhesion formation.

## INTRODUCTION

E-cadherin is a cell adhesion molecule required for epithelial tissue integrity (Jeanes et al., 2008). E-cadherin expression is down-regulated in many cancers, resulting in loss of cell adhesion and a switch from a benign epithelial state to an invasive mesenchymal phenotype (Thiery et al., 2009). However, many types of metastatic cancer, including pancreatic, retain E-cadherin-mediated cell-cell junctions (Gaida et al., 2012; Torres et al., 2013) and can migrate through a process known as collective invasion (Friedl et al., 2012). This suggests that mis-regulation of E-cadherin dynamics may play a role in promoting metastasis, and that better understanding of E-cadherin dynamics could lead to a better understanding of cancer cell invasion and new approaches to anti-invasive therapy (Serrels et al., 2009).

E-cadherin forms 3 primary structural interactions within adherens junctions. First, E-cadherin forms adhesive trans-dimers through a process termed strand swapping, in which the amino-terminal tryptophan of one partner is inserted into a hydrophobic pocket within the EC1 domain of the other (Boggon et al., 2002; Harrison et al., 2011). Second, E-cadherin can form lateral cis-oligomers, which are stabilized by hydrophobic interaction between V81 and L175 of adjacent monomers (Harrison et al., 2011). Finally, the cytoplasmic tail of E-cadherin can bind to β-catenin, which is structurally linked to the cortical actin meshwork via association with alpha-catenin. (This indirect linkage will be referred to here as “interaction” or “association” with the actin cytoskeleton) (Desai et al., 2013; Zaidel-Bar, 2013). E-cadherin clusters appear to be important functional units of adhesion. They have been widely observed at cell-cell junctions and studied at the micro-scale using diffraction limited optics (Adams et al., 1998; Hong et al., 2010; Kametani and Takeichi, 2007). Analysis using super-resolution microscopy has determined that E-cadherin cluster size scales continuously from the micro-scale down to the nano-scale; i.e. from hundreds of monomers per cluster down to 10 or less (Truong Quang et al., 2013). A model for the organization of E-cadherin within cell adhesions has been proposed based on structural analysis of extra-cellular domain crystals (Harrison et al., 2011). This model envisages a lattice of parallel strands of cis-oligomers oriented at 90° in opposing cell membranes and linked via trans-dimers. Recently super-resolution microscopy has shown that sub-regions of adhesive clusters contain the monomer packing density required to form the crystal lattice (Wu et al., 2015). The steps involved in adhesive cluster formation remain unclear, although the formation of clusters by tailless cadherin mutants has been taken as evidence that the first step in adhesion formation is the extra-cellular association of trans-dimers (Hong et al., 2010).

We have previously used fluorescence recovery after photo-bleaching (FRAP) both *in vitro* and *in vivo* to analyze the dynamics of E-cadherin-GFP (Ecad-GFP) during cell migration and in response to therapeutic intervention with the Src inhibitor Dasatinib (Serrels et al., 2009). This technique typically involves rapidly bleaching a small region of interest (ROI) at the midpoint of a cell-cell junction, and observing fluorescence recovery into the bleached region using time-lapse microscopy. Although FRAP is sometimes analyzed qualitatively (Hong et al., 2011, 2013), simple quantification of FRAP is achieved by fitting an exponential curve to the time series of fluorescence intensity measurements from an ROI (Sprague and McNally, 2005) and more complex analysis yielding insight into reaction kinetics can be achieved by fitting recovery curves to a reaction-diffusion equation (Thoumine et al., 2006).

Exponential analysis provides insight into two aspects of E-cadherin dynamics: the proportion of E-cadherin free to move within the plasma membrane and the rate at which it moves. The proportion of E-cadherin free to move is quantified by the mobile and immobile fractions (F_m_ and F_i_, where F_m_+F_i_=100%). F_i_ is an estimation of the amount of cadherin trapped in a cell junction, however single molecule tracking experiments on free cell surfaces have shown that E-cadherin can be non-specifically trapped in ‘membrane fence’ compartments (Iino et al., 2001; Kusumi et al., 1993). The relative contribution of non-specific interactions to immobilization of E-cadherin within cell-junctions is not known. The rate of E-cadherin movement may be quantified by the half-time of recovery (T_1/2_) (Lippincott-Schwartz et al., 2001), and can be influenced by many factors including membrane compartmentalization (Suzuki et al., 2005) and the presence of interactions with stationary binding partners (Sprague and McNally, 2005). If binding interactions are absent or weak, T_1/2_ is an estimation of the effective diffusion rate of E-cadherin. However, if binding interactions form quickly and last long, T_1/2_ can be used to estimate the molecular dissociation rate (Bulinski et al., 2001). Although FRAP has been widely used to study E-cadherin dynamics (de Beco et al., 2009; Harrison et al., 2011; Hong et al., 2010; Nanes et al., 2012; Ratheesh et al., 2012; Serrels et al., 2009; Yamada et al., 2005), it is unclear which molecular interactions of E-cadherin determine the FRAP parameters of F_m_, F_i_, and T_1/2_. This severely limits the interpretation of E-cadherin FRAP data.

In the present study we have used a pancreatic cancer model (Morton et al., 2010a,b) to systematically investigate the mobility of E-cadherin in cell-cell junctions using mutant analysis, chemical cross-linking, co-culturing of expression level variants, and super-resolution microscopy. We have identified four distinct populations of E-cadherin based on their differential inclusion into adhesive structures and mobility as quantified by FRAP. Our data support a model in which the first interaction of adhesion formation is association with the actin cytoskeleton, and allow us to draw conclusions about the dynamic composition of cis-oligomers in cadherin clusters.

## RESULTS

### E-cadherin localizes in sub-resolution clusters in pancreatic cancer cells

**Fig. 1.**
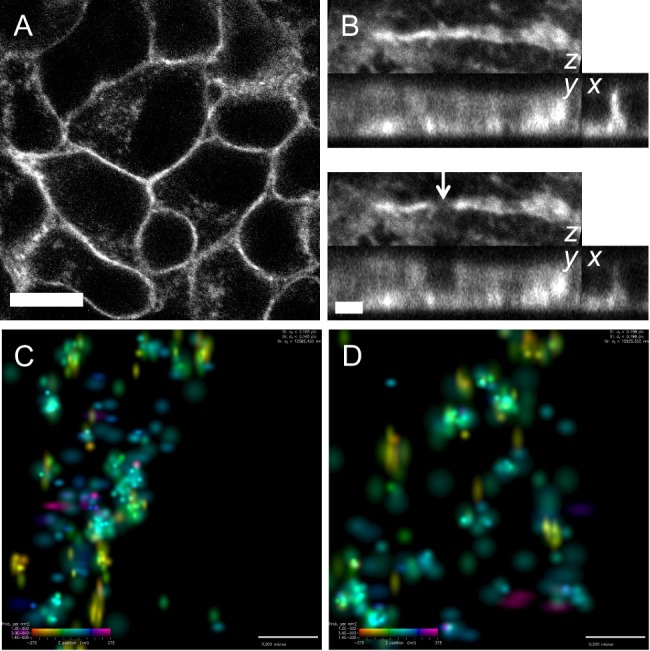
**E-cadherin localizes in nano-scale clusters.** (A) Ecad-GFP localization appears continuous in the junctions of pancreatic cancer cells when imaged using confocal microscopy. Bar: 10 µm. (B) Fixed cells were imaged by serial confocal sectioning before and after ROI photobleaching (top and bottom panels respectively, arrow in bottom panel highlights region of photobleaching). 3D data sets were reconstructed as projections to visualize the cell junction along the *z*, *y*, and *x*-axes. The *x*-axis projection was cropped to the photobleached region, and shows that junctions were vertical and did not undercut adjacent cells. Bar: 2 µm. (C) At higher resolution it was apparent that E-cadherin was localized in clusters. (D) Examination of cells expressing lower levels of Ecad-GFP revealed that E-cadherin clusters maintained the same average size and number of monomers per cluster, but that the spacing between clusters increased. In 3D-STORM images the *z*-position of each molecule is color-coded and its intensity indicates positional accuracy according to the look-up table in each panel. Color bar in lower left of panels indicates the *z*-position range from −375 to +375 nm (left to right) and probability per nm^2^ from 1.2×10^−2^ to 1.4×10^−5^ (top to bottom). Bar in C,D: 200 nm.

To investigate the localization and dynamics of E-cadherin in pancreatic cancer cells, PDAC tumor cells isolated from Pdx1-Cre, LSL-KRas^G12D/+^, Trp53^LoxP/+^ mice (Morton et al., 2010b) were stably transfected with GFP-chimeras of wild-type E-cadherin or mutants. PDAC cells were fixed and serial confocal sections acquired in order to visualize the 3-dimensional structure of junctions in these cells (Fig. 1A and B). Reconstruction of 3D data sets acquired using diffraction limited optics revealed a relatively homogenous distribution of Ecad-GFP in the plasma membrane. Cell junctions appeared vertical and did not significantly undercut adjacent cells, indicating that they were mature and likely to be under tension (Kametani and Takeichi, 2007). In order to probe the organization of E-cadherin at higher resolution, we used Stochastic Optical Reconstruction Microscopy (STORM). At low magnification the localization of E-cadherin within cell-cell junctions obtained using STORM was similar to that obtained using confocal microscopy (supplementary material Fig. S1). However, at higher magnification it was apparent that E-cadherin was localized in clusters (Fig. 1C and D). Mean shift analysis of E-cadherin distribution revealed the average number of monomers per cluster to be 10.32±7.72 within an average diameter of 131.0±45.6 nm. The average distance between clusters was 205.9±113.5 nm. To examine the effect of cadherin expression level on cluster parameters, cells were sorted by FACS (supplementary material Fig. S2) for low GFP fluorescence and analyzed using STORM. In this case the average cluster diameter and number of monomers per cluster were similar, however the average distance between clusters was 65% greater (supplementary material Fig. S1B and Table S1). These data show that despite its homogenous appearance in the confocal microscope, E-cadherin was organized in clusters on the sub-diffraction limited scale. Furthermore, PDAC cells responded to different expression levels of E-cadherin by varying the spacing between same-sized clusters, rather than maintaining the spacing between differently sized clusters.

### E-cadherin is immobilized through adhesive and non-adhesive interactions at cell junctions

**Fig. 2.**
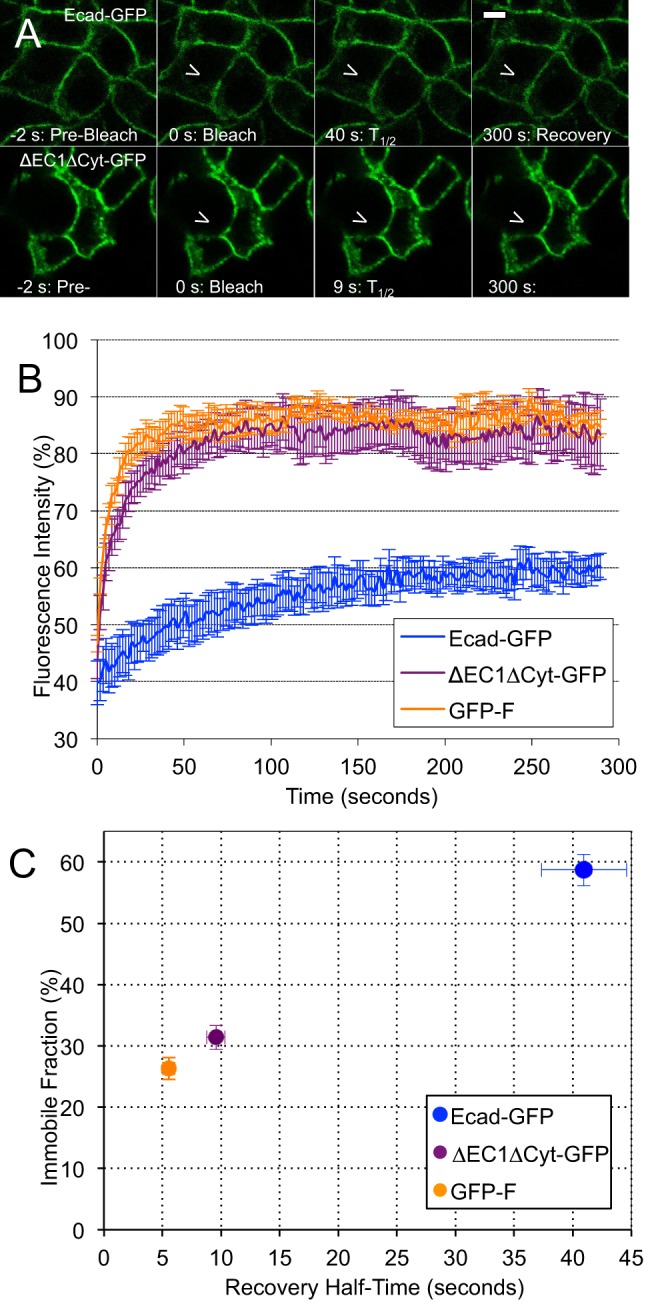
**Ecad-GFP is immobilized through adhesive and non-adhesive interactions at cell junctions.** (A) FRAP analysis of PDAC cells expressing either Ecad-GFP (top row) or a mutant unable to form cis-, trans-, or actin interactions (ΔEC1ΔCyt-GFP, bottom row). Bar: 5 µm. (B) Average fluorescence recovery curves for Ecad-GFP, ΔEC1ΔCyt-GFP and GFP-F. (C) The half-time of recovery (T_1/2_) and immobile fraction (F_i_) were derived from exponential functions fitted to individual FRAP curves, error bars represent s.e.m. Note that although the graph shows F_i_ and T_1/2_, T_1/2_ is actually a property of F_m_. See supplementary material Table S2 for a complete list of FRAP parameters.

The immobile fraction determined by FRAP is typically used to estimate the amount of E-cadherin involved in cell-cell adhesion. However, single particle tracking experiments at the free cell surface have previously shown that a fraction of E-cadherin is immobilized within the plasma membrane by non-specific trapping of molecules in “corrals” formed by the cortical membrane cytoskeleton (Kusumi et al., 1993; Sako et al., 1998). To estimate the contribution of non-specific trapping to the immobilization of E-cadherin at cell-cell junctions, rather than the free cell surface, we compared the mobility of Ecad-GFP with the non-functional mutant ΔEC1ΔCyt-GFP (Fig. 2). This mutant lacks both the EC1 and cytoplasmic domains, and is therefore unable to form cis-, trans-, or actin interactions. FRAP analysis revealed that the immobile fraction of ΔEC1ΔCyt-GFP was 31.38±1.96% (*n*=39 junctions), compared to 58.69±2.57% (*n*=24) for Ecad-GFP (Fig. 2C, for a complete list of FRAP parameters see supplementary material Table S2). As a control we analyzed GFP localized to the inner leaflet of the plasma membrane via the membrane targeting sequence from HRas (GFP-F) (Serrels et al., 2009), which can not bind to any other proteins. Interestingly, the level of non-specific trapping was similar to ΔEC1ΔCyt-GFP (F_i_=26.34±1.82%, *n*=10, Fig. 2C). Similar results were obtained using GFP targeted to the plasma membrane via the PH domain of AKT and the neuromodulin palmitoylation signal (MEM) (supplementary material Fig. S3A). To confirm that 50% photo-bleaching as used in these experiments did not result in non-specific crosslinking of GFP-tagged molecules, photobleaching of GFP-F and ΔEC1ΔCyt-GFP was performed at 25% bleaching intensity and similar results were obtained (supplementary material Fig. S3B). To confirm the involvement of actin in the non-specific trapping assessed by FRAP, PDAC cells expressing GFP-F were treated with latrunculin A, which resulted in 40% reduction in the immobile fraction (supplementary material Fig. S3C). These data suggest that approximately half of E-cadherin trapped in cell-cell junctions is immobilized through non-specific interactions. The molecular weight and geometry of ΔEC1ΔCyt-GFP are similar to Ecad-GFP and should therefore indicate the rate of E-cadherin diffusion in the absence of any binding interactions. The recovery rate of ΔEC1ΔCyt-GFP was much faster than Ecad-GFP but statistically equivalent to GFP-F, suggesting that the mobility of Ecad-GFP is not limited by diffusion. From these data we conclude that E-cadherin is immobilized at cell-cell junctions through two mechanisms: by specific interactions mediated by the EC1 and/or cytoplasmic domain, and by non-specific interactions such as the cortical membrane fence. It is also apparent that the recovery rate of Ecad-GFP is much slower than would be expected for a process limited by the rate of diffusion alone.

### Inclusion of E-cadherin into stationary clusters requires cis-, trans-, and cytoplasmic interactions

**Fig. 3.**
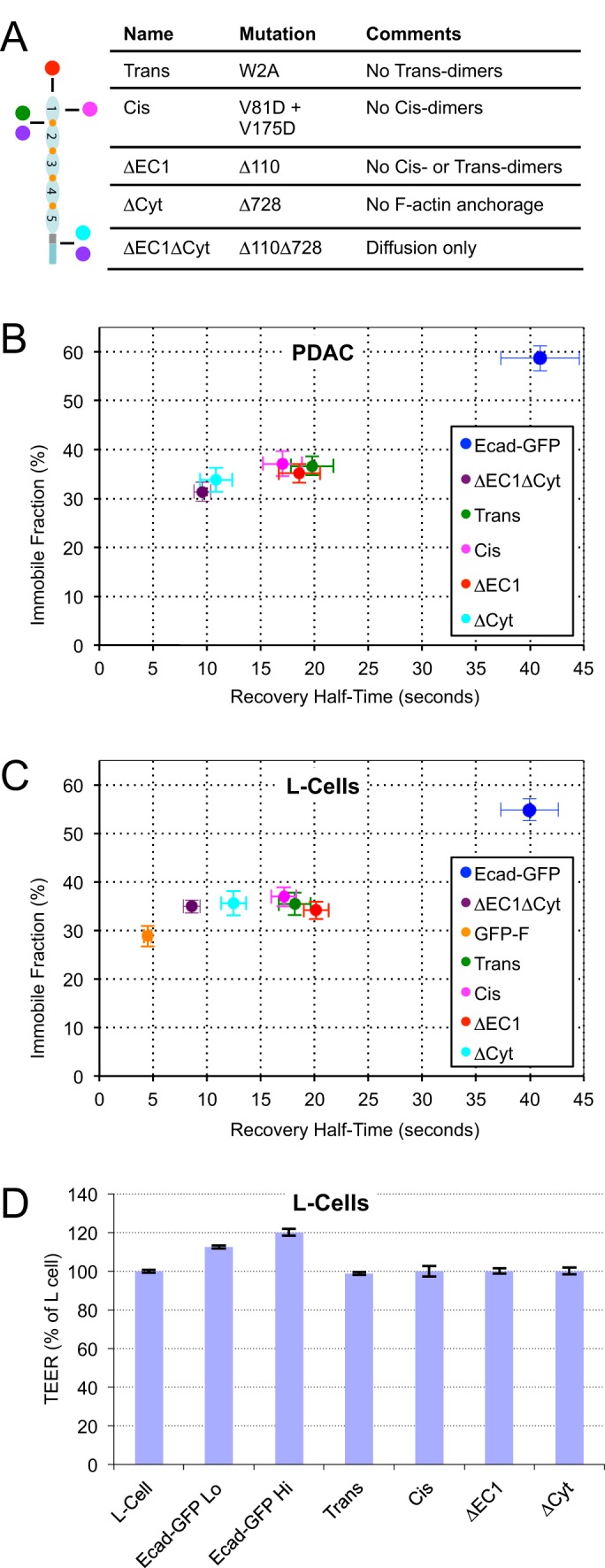
**Inclusion of E-cadherin into stationary clusters requires cis-, trans-, and cytoplasmic interactions.** (A) Schematic diagram and table of mutants. (B,C) FRAP analysis of wild-type Ecad-GFP and mutants expressed in PDAC (B) and L-cells (C). Deletion of any single interaction reduces F_i_ from the level of wild-type E-cadherin (∼60%) to the level of the non-interacting mutant ΔEC1ΔCyt (∼30%), indicating that all three interactions, cis-, trans-, and cytoplasmic, are required for inclusion of Ecad-GFP into stationary adhesive clusters. Those mutants retaining actin association (trans-, cis-, and ΔEC1 mutants) recover more slowly than mutants lacking the cytoplasmic domain (ΔEC1ΔCyt, and ΔCyt). Retention of cis- and trans- interactions by the ΔCyt mutant did not significantly slow its recovery compared to the ΔEC1ΔCyt mutant. Values for Ecad-GFP and ΔEC1ΔCyt are included from Fig. 2C for comparison; see supplementary material Table S2 for list of all FRAP parameters. (D) TEER measured in L-cells expressing low and high levels of wild-type E-cadherin, and E-cadherin mutants. Note that none of the mutants were able to increase the effective cell adhesion strength as assessed by electrical resistance above the level of L-cells, which did not express E-cadherin. *N*=3 for each condition; error bars represent s.e.m.

Comparison of wild-type E-cadherin with the non-functional mutant suggested that up to half of the stationary E-cadherin in cell junctions was immobilized through specific interactions. We therefore wanted to investigate the relative contributions of cis-, trans-, and actin interactions to the immobilization of E-cadherin within cell junctions. To do this, we made mutants defective for trans- [W2A (Kitagawa et al., 2000)] and cis- [V82D+V175D (Harrison et al., 2011)] interactions, as well as deletion mutants lacking the EC1 (Δ110) and cytoplasmic (Δ110) domains, expressed them individually in PDAC cells in conjunction with endogenous E-cadherin, sorted them for expression level, and analyzed their mobility using FRAP (Fig. 3A,B). Strikingly, we found that disruption of any single interaction was sufficient to reduce the immobile fraction of E-cadherin to the level of the non-functional mutant ΔEC1ΔCyt-GFP. This indicates that all three interactions (cis-, trans-, and actin) are required for the specific immobilization of E-cadherin at cell-cell junctions. Furthermore the mutants all recovered more quickly than Ecad-GFP, and clustered into two groups based on their mobility rates (Fig. 3B). The cytoplasmic deletion mutant ΔCyt-GFP recovered at the rate of ΔEC1ΔCyt-GFP, demonstrating that cis- and trans- interactions mediated by the EC1 domain did not restrain its diffusion. In contrast mutants retaining the cytoplasmic tail recovered significantly more slowly. However there were no differences among tail-containing mutants on the basis of individual or combined EC1-mediated interactions. These data show that cytoplasmic interactions occurred without EC1 interactions but not visa-versa, and indicate that the slow recovery rate of wild-type Ecad-GFP depends on the ability to form cis-, trans-, and actin interactions. Similar results were obtained in L-cells lacking endogenous, E-cadherin (Fig. 3C). In order to assess the functional consequences of mutant E-cadherin expression, L-cells were grown on permeable membranes and the effective cell adhesion strength of confluent monolayers assessed using trans-epithelial electrical resistance (TEER). This analysis showed that wild-type E-cadherin was able to increase the adhesion strength of L-cells in a dose dependent manner. In contrast, none of the E-cadherin mutants were able to elevate the electrical resistance of L-cells, supporting our conclusion that the ∼35% immobile fraction retained by these mutants had no adhesive function.

### E-cadherin expression levels influence monomer dynamics

**Fig. 4.**
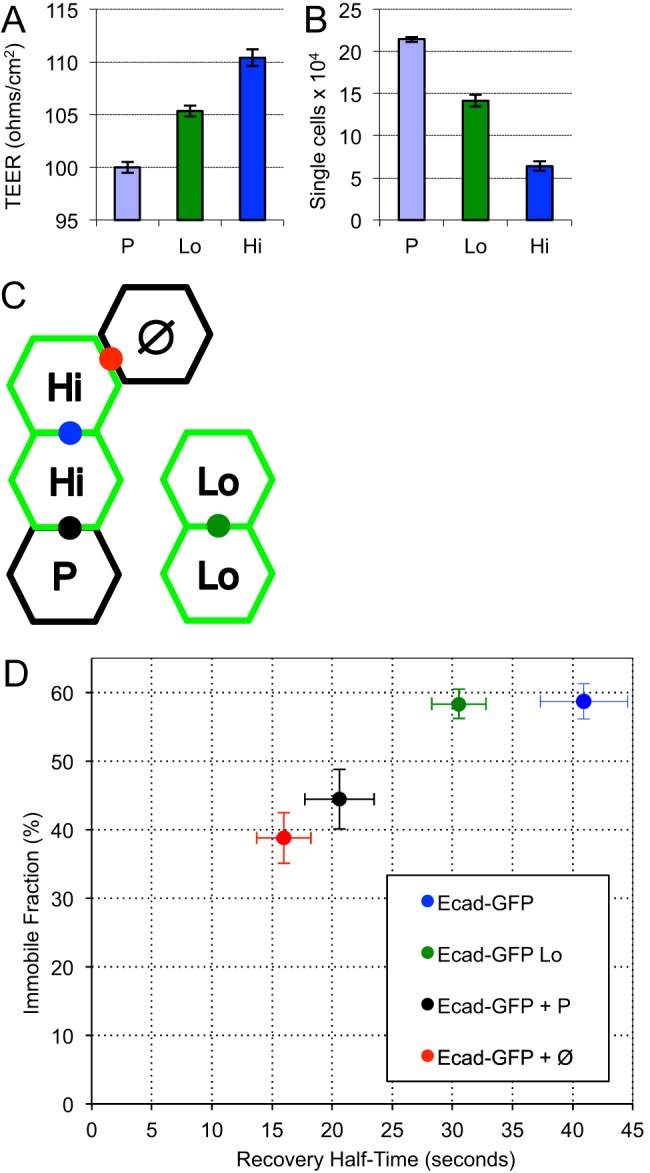
**E-cadherin expression level affects monomer dynamics.** (A,B) Estimation of junctional integrity and cell adhesion strength using TEER (A) and Dispase (B) assays. *N*=3 for each; error bars represent s.e.m. (C) Schematic diagram of different types of cell junctions assayed by FRAP, showing junctions between Ecad-GFP Hi cells (Hi), between Ecad-GFP Lo cells (Lo), between Ecad-GFP Hi cells and the parental cell line (P) expressing endogenous E-cadherin, and between Ecad-GFP Hi cells and L cells (Ø) expressing no E-cadherin. (D) F_i_ of Ecad-GFP is the same in Ecad-GFP Hi and Ecad-GFP Lo cells (blue and green circles respectively), however T_1/2_ is shorter for Ecad-GFP Lo cells. The level of Ecad-GFP expression in the neighboring cell of a junction also affects E-cadherin dynamics. When FRAP is measured between cells expressing high levels of Ecad-GFP and no Ecad-GFP (black circles) both T_1/2_ and F_i_ are significantly reduced. F_i_ and T_1/2_ are further decreased in the absence of trans-dimer formation (red circle). Value for Ecad-GFP is included from Fig. 2C for comparison; see supplementary material Table S2 for list of all FRAP values. Error bars represent s.e.m.

Previous work has shown that the E-cadherin expression level influences cell adhesion strength (Steinberg and Takeichi, 1994). In order to explore the relationship between E-cadherin expression level and protein mobility, PDAC cells expressing high (Hi) and low (Lo) levels of Ecad-GFP were first analyzed using TEER and resistance to dispase treatment. As expected, the effective cell adhesion strength of confluent PDAC cells increased with E-cadherin expression level (Fig. 4A). In agreement with this, dispase assay analysis confirmed that cell adhesion strength increased with increasing E-cadherin expression (Fig. 4B). Despite their differences in adhesion strength, FRAP analysis revealed that the immobile fraction of E-cadherin was equal for both Hi and Lo expressing PDAC cells (Fig. 4D). This indicates that although Hi-expressing cells engage more E-cadherin overall (as judged by cell adhesion strength) the proportion of free and immobilized E-cadherin remains constant, as would be expected for a process governed by simple dynamic equilibrium. Thus, the immobile fraction alone does not serve as an indicator of cell adhesion strength. In contrast the recovery rate was faster for the Lo-expressing cells (Fig. 4D). Similar results were obtained with L-cells (supplementary material Fig. S4).

Cancer cells typically down-regulate E-cadherin expression during epithelial to mesenchymal transition, leading to an imbalance in E-cadherin levels between adjacent cells. To investigate the dependency of E-cadherin mobility on the expression level of the cell junction partner, Ecad-GFP Hi cells were co-cultured with the parental PDAC line (Fig. 4C and D). The total level of E-cadherin expression is lower in the parental line because it does not express Ecad-GFP in addition to endogenous unlabeled E-cadherin (supplementary material Fig. S2C). As such, this FRAP experiment only reports on behavior within the Ecad-GFP Hi cells. We found that both F_i_ and T_1/2_ were significantly reduced compared to FRAP performed between two Ecad-GFP Hi cells (Fig. 4D and supplementary material Table S2), demonstrating that formation of adhesive complexes is limited by the availability of E-cadherin in each partner of a cell junction. As an extreme example of unbalanced E-cadherin expression levels between cells, Ecad-GFP Hi cells were co-cultured at high density with L-cells, which do not express any cadherin. Interestingly, the mobile fraction and mobility rate of Ecad-GFP at sites of L-cell contact were statistically equivalent to the E-cadherin trans mutant analyzed in PDAC or L-cell junctions. In all three cases the protein under investigation was unable to form trans-associations with a neighboring cell (compare red circles in Fig. 4D with Fig. 3B and C, see also supplementary material Table S2). The recovery rate of Ecad-GFP at PDAC-L-cell junctions was slower than the rate of tailless mutants in PDAC or L-cells, but similar to the rate of mutants which retained the cytoplasmic tail. These data confirm a role for the cytoplasmic tail in restraining the diffusion of E-cadherin.

### Crosslinking reveals free and transiently associated mobile populations

**Fig. 5.**
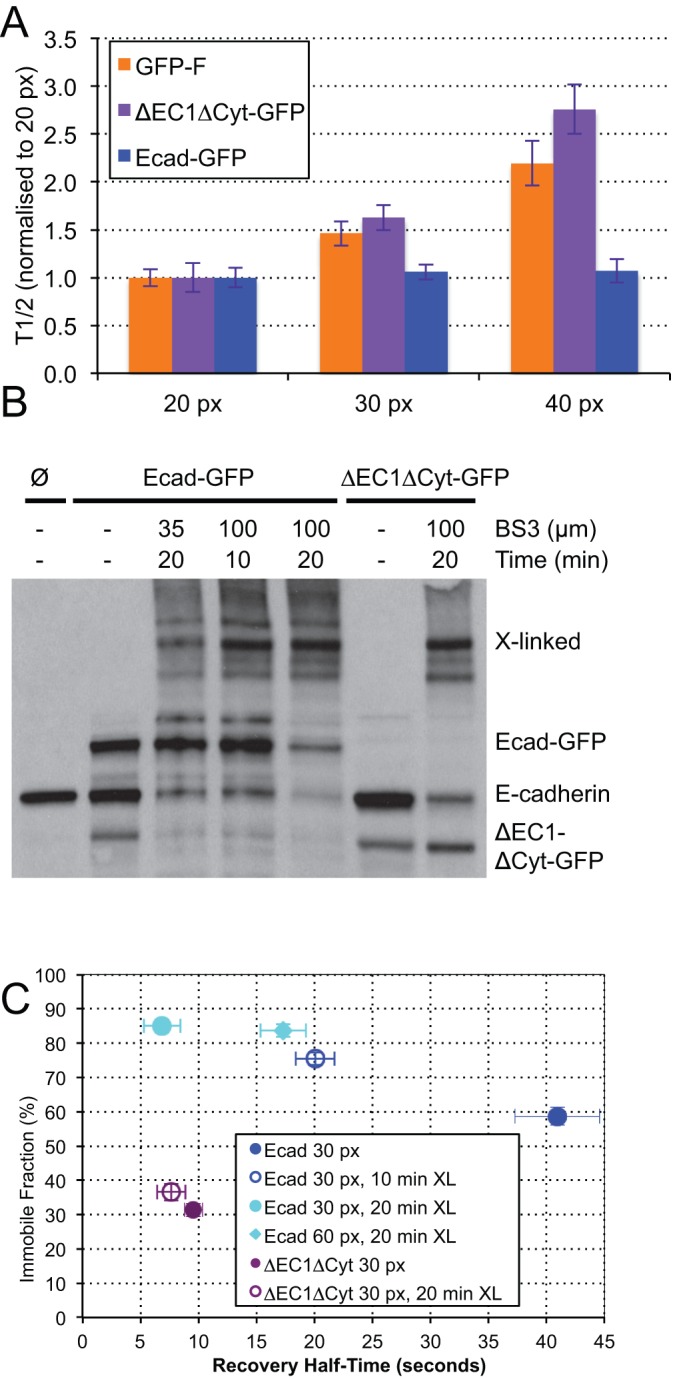
**Crosslinking reveals fast and slow recovering populations.** (A) FRAP analysis of the effect of ROI size on T_1/2_. (B) Western blot probed with anti-E-cadherin antibody demonstrating effects of BS3 treatment. PDAC cells express endogenous E-cadherin, whereas Ecad-GFP cells express endogenous and GFP-labeled protein. Treatment for 20 min in 35 µM BS3 or 10 min in 100 µM BS3 inefficiently cross-linked E-cadherin, whereas 20 min in 100 µM BS3 cross-linked the majority of endogenous and GFP-labeled E-cadherin on the cell surface, as evidenced by the reduction in monomeric E-cadherin. Treatment for 20 min in 100 µM BS3 was unable to cross-link ΔEC1ΔCyt-GFP. The band in lane 2 which runs in the position of ΔEC1ΔCyt-GFP is most likely a degradation product of E-cadherin because it does not blot for GFP. (C) FRAP analysis showing the effects of cross-linking and ROI size. Cross-linking of ΔEC1ΔCyt-GFP had no effect on its mobility. In contrast, the F_i_ of Ecad-GFP cross-linked with BS3 for 20 min increased from 60% to 85% and the T_1/2_ decreased from 40 s to 7 s. To confirm that the recovery of Ecad-GFP had become diffusion coupled following cross-linking, the ROI diameter was doubled from 30 to 60 pixels, which significantly increased the recovery half-time. Cross-linking PDAC cells for 10 min in 100 µm BS3 only partially shifted the FRAP parameters towards diffusion uncoupled recovery. Values for Ecad-GFP and ΔEC1ΔCyt are included from Fig. 2C for comparison; see supplementary material Table S2 for list of all FRAP values. A,C: error bars represent s.e.m.

Previous work has shown that the mobility of E-cadherin is primarily limited by its slow turnover in adhesive clusters (Thoumine et al., 2006). However we observed that the recovery rate of E-cadherin was sensitive to the spacing between clusters, suggesting that recovery could be diffusion limited. To determine whether recovery was diffusion or reaction limited we varied the size of the ROI used for bleaching and analysis (Nanes et al., 2012; Sprague and McNally, 2005). In the case of pure diffusion T_1/2_ should increase with increasing ROI size, whereas the rate of molecular turnover does not depend on the size of the bleached region. GFP-F, ΔEC1ΔCyt-GFP, and Ecad-GFP were analyzed using ROIs of 20, 30, and 40 pixels in diameter (Fig. 5A). As expected, the recovery time of both GFP-F and ΔEC1ΔCyt-GFP increased with increasing ROI diameter, confirming that the mobility of these molecules is limited by diffusion. In contrast, the recovery time of Ecad-GFP remained constant with increasing ROI diameter, indicating that its mobility was limited by molecular turnover.

To establish the size of the dynamic population interacting with stationary complexes, we performed cross-linking experiments using the cell-impermeable homo-bifunctional cross-linker Bis[sulfosuccinimidyl] suberate (BS3). Based on the short linking radius of this compound (11.4 Å), it is expected only to cross-link molecules in direct proximity. We first established conditions in which E-cadherin was effectively cross-linked by BS3 (Fig. 5B). 20 min of treatment with 35 µM BS3 resulted in some cross-linking of E-cadherin, however 20 min of 100 µM BS3 treatment resulted in the majority of both E-cadherin and Ecad-GFP monomers being cross-linked. In contrast ΔEC1ΔCyt-GFP was not cross-linked by BS3. These data indicate that the majority of E-cadherin is able to self-associate in the plasma membrane whereas ΔEC1ΔCyt-GFP is non-interactive. We next examined the mobility of Ecad-GFP in PDAC cells treated with BS3 (Fig. 5C). Interestingly, we found that the mobile fraction of Ecad-GFP was reduced from 40% to 15% following BS3 treatment and its half time of recovery was dramatically reduced from ∼40 s to 7 s, similar to the rate of membrane targeted GFPs. The rapid mobility of the Ecad-GFP which remained following BS3 treatment suggested that this remaining mobile fraction recovered by molecular diffusion unrestrained by binding interactions. To confirm this we increased the size of the analysis ROI for cross-linked Ecad-GFP to 60 pixels in diameter, and observed a corresponding increase in T_1/2_. In contrast to Ecad-GFP, and in agreement with western blot analysis, the mobility of ΔEC1ΔCyt-GFP assessed by FRAP was unaffected by BS3 cross-linking. These data reveal that the 40% mobile fraction of Ecad-GFP in cell junctions is comprised of two components: a larger slow component which can be cross-linked by BS3 (∼25%) and a smaller fast component which can not (∼15%).

## DISCUSSION

The primary adhesive bond between adjacent cells is thought to be the strand-swapped trans-interaction (Harrison et al., 2011), which transmits tension to the actin cytoskeleton via association of the tail domain with β-catenin (Barry et al., 2014). Cis-interactions, while not directly adhesive, are thought to cooperate in adhesion by promoting clustering (Hong et al., 2013). In addition to these interactions, X-dimers are through to promote the transition in and out of the strand-swapped state (Hong et al., 2011; Rakshit et al., 2012). The functional relevance of the many possible combinations of E-cadherin interactions remains unclear. A central issue in understanding the biochemistry of cadherin interactions is that association constants measured *in vitro* are orders of magnitude weaker that those measured between cells (Leckband and Sivasankar, 2012). This may indicate that, like Velcro, strong cell adhesions can result from many weak interactions. Alternatively, it may be that biochemical investigations fail to capture essential properties of cadherin interactions such as their geometry in the cell membrane, the architecture of the cortical cytoskeleton, the influence of force, or the role of associated proteins such as alpha-catenin and vinculin. Therefore it remain essential to investigate cadherin dynamics *in situ*.

**Fig. 6.**
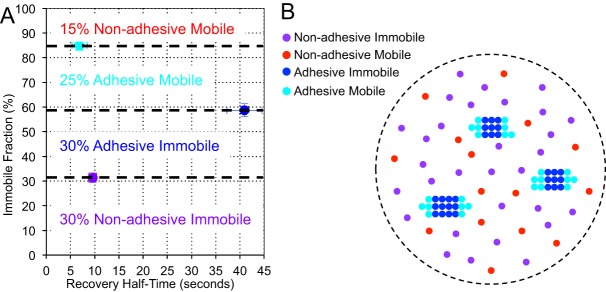
**Schematic diagram showing distribution and dynamics of four E-cadherin populations within the ROI of a FRAP experiment.** Non-adhesive immobile monomers (purple) are trapped through non-specific interaction with the cortical cytoskeleton. Non-adhesive mobile monomers (red) are able to move but do not bind to complexes. Adhesive immobile monomers (blue) remain stationary, possibly by virtue of being trapped within cis-strands. Adhesive mobile monomers (cyan) are in dynamic equilibrium with stationary complexes and alternate between transient binding and diffusion.

FRAP has been widely used to study the dynamics of E-cadherin in cell adhesion (Harrison et al., 2011; Hong et al., 2011, 2013; Lambert et al., 2007; Nanes et al., 2012; Ratheesh et al., 2012; Serrels et al., 2009; Thoumine et al., 2006; Yamada et al., 2005), yet the molecular interactions which determine the mobility and adhesive properties of E-cadherin remain poorly defined. Here we have systematically analyzed the determinants of E-cadherin mobility and find that the mobile and immobile fractions identified by FRAP each contain adhesive and non-adhesive sub-populations. Our data allow us to distinguish 4 populations of E-cadherin in cell-junctions (Fig. 6A). The first population is represented by the 30% immobile fraction of ΔEC1ΔCyt-GFP which is trapped through non-specific interactions, presumably corralled through interactions with the cortical membrane fence (Iino et al., 2001). ΔCytΔEC1-GFP is critical for the interpretation of our results, establishing both the level of non-specific trapping experienced by cadherin molecules in cell junctions, and the rate of cadherin mobility in the absence of cis-, trans-, or actin interactions. The second population is the 30% of wild-type E-cadherin specifically immobilized through the cumulative effect of cis-, trans-, and actin interactions. The third population is the 25% mobile fraction which can be trapped by BS3 cross-linking. The slow recovery rate of this mobile fraction compared to any of the deletion mutants depends on the cumulative effect of cis-, trans-, and actin interactions. The slow turnover rate we observe is consistent with elegant studies of cadherin mobility performed by the Choquet and colleagues (Thoumine et al., 2006), which showed that the mobility of E-cadherin is primarily limited by the slow turnover of monomers in dynamic equilibrium with stationary clusters. The dependence of monomer immobilization and slow turnover on all three interactions indicates their contribution to cell adhesion. Together, the adhesive mobile and immobile fractions comprise 55% of total E-cadherin in PDAC cell-cell junctions. The fourth population is the 15% mobile fraction which cannot be cross-linked by BS3 and recovers at the rate of ΔEC1ΔCyt-GFP. Interestingly, this population does not appear to engage in tail interactions with the cytoskeleton. The association between E-cadherin and β-catenin is primarily regulated through phosphorylation of β-catenin (Nelson and Nusse, 2004). However the affinity of E-cadherin for β-catenin also depends on phosphorylation of multiple serine residues between amino-acids 833-862 of E-cadherin (Lickert et al., 2000). Thus the 15% non-adhesive mobile fraction we observe could represent e-cadherin monomers unable to associate with β-catenin. While the relative proportions of these four populations may be different for different cell types, we expect that similar functional and dynamic populations will exist in all epithelial cells.

Note that other interpretations of the BS3 cross-linking experiments are possible. For example, if there were a limited number of E-cadherin binding sites available it may be that cross-linking could block them, resulting in increased mobility of the non-cross-linked molecules because the cross-linked molecules stably occupy actin filaments. In this respect, the availability of E-cadherin binding sites within cell-cell adhesions is an important parameter warranting further study.

The mechanism by which cells regulate trans-dimer formation, and thereby cell adhesion, remains unclear. Our data are consistent with a model in which individual interactions are weak, and multiple interactions are required in order to stabilize E-cadherin within the adhesive fraction. Our data suggest that regulation of any single interaction, cis-, trans-, or actin, is sufficient to drive E-cadherin in or out of cell adhesions. Of these three interactions, only the intra-cellular interaction is known to be directly regulated. Recent work has shown that cytoplasmic domain deletion mutants of E-cadherin can form patches at sites of cell-cell contact, which approximate the localization of wild-type E-cadherin seen in the light microscope (Harrison et al., 2011; Hong et al., 2010; Ozaki et al., 2010). The ability of these mutants to cluster has suggested an “outside-in” mechanism for junction assembly in which extra-cellular interactions precede intra-cellular interactions (Brasch et al., 2012). We did not observe the clustering of tailless mutants, and they were unable to increase the adhesion strength assessed by TEER of L-cells. Instead, we observed that cytoplasmic interactions significantly slowed the diffusion of wild-type and mutant E-cadherin, whereas cis- and trans- interactions did not. Our data thus suggest an ‘inside-out’ model in which cytoplasmic interactions precede extra-cellular interactions during junction assembly, and regulation of actin association alone could be sufficient to regulate adhesive complex formation.

Using conventional diffraction limited optics, we found the distribution of E-cadherin to be relatively homogenous. However, in agreement with recent results from epithelial junctions in *Drosophila* embryos (Truong Quang et al., 2013), we found E-cadherin to be organized in clusters at the nano-scale. Our data on cadherin cluster size and spacing are consistent with recent results obtained in Eph4 cells (Wu et al., 2015). Interestingly, we found that in cell expressing more E-cadherin, the average cluster size remained constant whereas the spacing between clusters decreased. Increased spacing between adhesive clusters would appear to offer an explanation for the rapid recovery observed in Ecad-GFP Lo cells compared to Hi cells, on the basis of longer diffusion runs between binding events. However, examination of ROI size supported molecular binding, not diffusion, as the factor limiting E-cadherin mobility. Further investigation into the effects of expression level on cadherin dynamics is required.

How are the mobile and immobile fractions organized within adhesive clusters? It is possible that these fractions represent distinct populations of adhesive clusters, or that mobile and immobile behavior results through differential regulation of E-cadherin binding. However, differences in mobility could more simply result from the arrangement of E-cadherin monomers within clusters according to the model of Harrison et al. (2011). According to this model, E-cadherin monomers are arranged in parallel strands of cis-associated monomers. In this arrangement monomers at the ends of cis-strands would be more easily exchanged than monomers in the middle, and monomers in the middle would therefore effectively be trapped (Fig. 6B). We propose that the adhesive mobile fraction consists of cadherin monomers capping the ends of cis-oligomer strands, thereby trapping the adhesive immobile fraction within the interior of the strand. This proposal does not depend on differential regulation of cadherin monomers to result in adhesive mobile and immobile populations of E-cadherin. The roughly 1:1 ratio of mobile to immobile adhesive monomers we observed should serve as a useful boundary condition for molecular simulations of cadherin dynamics.

In summary, our data provide a framework for understanding E-cadherin dynamics at cell-cell junctions based on specific and non-specific molecular interactions (Fig. 6B). By comparing the disruption of all three E-cadherin interactions, we have revealed aspects of cadherin behavior which could not be deduced from any single comparison with wild-type protein. Our data support a model for E-cadherin based junction formation in which dynamic monomers in equilibrium with adhesive clusters both contribute to cell-cell adhesion, and adhesion formation begins via association of E-cadherin with the actin cytoskeleton.

## MATERIALS AND METHODS

### Plasmids

The GFP-F plasmid was obtained from Clontech and the pcDNA3-Ecadherin-GFP plasmid was a gift from Jennifer Stow (The University of Queensland, Australia). pAcGFP1-Mem Hyg, the N-terminal membrane targeting signal of neuromodulin (also known as GAP-43) was from Clontech. The PH-domain from human AKT1 was a gift from Tamas Balla (Varnai et al., 2005). All E-cadherin GFP mutants were prepared from the pcDNA3-Ecadherin-GFP plasmid using the Quick Change Lightning site directed mutagenesis kit (Agilent Technologies) according to the manufacturer's protocol. For deletion of the cytoplasmic domain (ΔCyt), NotI restriction sites were inserted on each side of the cytoplasmic domain and NotI restriction enzyme was used to cut out residues 580 to 726. For Deletion of the EC1 domain (ΔEC1), two XhoI sites were introduced in the same way to excise the region encoding amino acids 2 to 109.

### Cell culture & transfection

PDAC cells were derived from pancreatic tumors harvested from Pdx1-Cre, LSL-KRas^G12D/+^, LSL-Trp53^R172H/+^ mice (Morton et al., 2010a,b) and L-cells were obtained from the ATCC (CRL-2648). Cells were maintained in DMEM supplemented with 10% FCS +2 mM L-glutamine +1% penicillin/streptomycin solution and sub-cultured weekly at a split ratio of 1:10. Both cell lines were transfected using Amaxa cell line nucleofector Kit V (Lonza). Transfected cells were selected using G418 sulphate solution at 0.7 mg/ml final concentration (Formedium). Cells were sorted by FACS into two populations of low and high expressing cells using a FACSAria.

### Cross linking and western blotting

PDAC cells were seeded confluently in 35 mm glass-bottomed microwell dishes (No. 1.5, MatTek), washed with HEPES/PBS buffer (20 mM HEPES, pH 7.6, 1 mM CaCl_2_, 150 mM NaCl). 100 µM BS3 crosslinker (Thermo Scientific) in water was added to the cells for 10 or 20 min then quenched by adding Tris-HCl (pH 7.5) to a final concentration of 20 mM. Cells were imaged for FRAP in cell culture media for 2 h, then lysed in Laemmli sample buffer (62.5 mM Tris-HCl, 10% Glycerol, 2% SDS, 5% Mercaptoethanol, 0.0025% Bromphenol Blue), sonicated, and analysed on NuPage Tris-acetate Gels (Life Technologies). Anti-E-cadherin antibody (BD Transduction Laboratories™, Cat.No.610182) was used for detection. Quantification of cadherin expression level was performed using a Licor Odessy with Licor anti-mouse IgG (Donkey) antibody conjugated to IRDye800. Tubulin was detected using Sigma T9026 anti-tubulin antibody.

### FRAP and data analysis

2.5×10^6^ PDAC cells (4×10^6^ for L cells) were plated onto glass-bottomed dishes and left to adhere overnight. Photo-bleaching experiments were performed using an Olympus FV1000 confocal microscope with SIM scanner. Cells were maintained at 37°C and imaged using the following settings: 4 μs pixel dwell time, 512×512 pixel resolution, 2% 488 nm laser power. For bleaching, a circular ROI with 30 pixel diameter (3 µm) was bleached to approximately 50% of its initial intensity using 35% 405 nm laser power, 20 μs/pixel dwell time for one frame. Images were captured every 1.6 s for 5 min. Individual recovery curves were exported into SigmaPlot (Systat Inc, London, UK) for exponential curve fitting. Data were fit using the following exponential functions: Y=Y_0_+a×(1−exp (−b×x)). The half-time of recovery was calculated using the formula T_1/2_=ln2/b, where b was obtained from the exponential curve fit. The immobile fraction was calculated as follows using values derived from the curve fit: F_i_=100×(1−a/(1−Y_0_)). Unpaired Student's *t*-test was used to test for statistical significance between groups of T_1/2_ and F_i_ providing the data passed tests for normality and variance; otherwise the Mann–Whitney Rank Sum Test was used. For detailed FRAP parameters and estimation of statistical significance see supplementary material Table S1; F_i_ values presented in the text are rounded to nearest 5%. Comparison of variance within and between experiments indicated that the primary source of variation was biological rather than experimental. Cells were treated with 1 µm Latrunculin A (Life Technologies L12370) for one hour prior to FRAP.

### Measurement of TEER and Dispase

For measuring TEER 3×10^5^ PDAC cells (or 6×10^5^ for L cells) were seeded overnight on transwell permeable supports (Costar). TEER was measured using an EVOM2 epithelial voltohmmeter with STX2 electrode (World Precision Instruments). For Dispase assays, the confluent cell monolayer was treated with 6 mg/ml DispaseII (Sigma) in PBS, the detached monolayer broken up by pipetting up and down and single cells counted after passing through a cell strainer (BD Falcon ,40 µm nylon) using a hemocytometer. Results are reported as number of single cells per 10,000 cells.

### Super resolution microscopy

#### Immunohistochemistry

Confluent cell monolayers on glass cover slips were used for immunostaining. Cells were fixed for 15 min with 4% paraformaldehyde (Electron Microscopy Sciences), washed, and then blocked and permeabilized using 10% FBS in DPBS with 0.1% Triton X-100, followed by 1 h incubation at room temperature with anti-Ecadherin produced in mouse (BD Biosciences) and 45 min incubation with Alexa Fluor 647 donkey anti-mouse (Life Technologies). After antibody labeling cells were finally fixed for 5 min in 3% PFA with 0.05% glutaraldehyde.

#### STORM image acquisition

STORM data acquisition was started with continuous imaging laser illumination (647 nm, 100 mW) at 30 frames per second, using a commercially available Nikon Elements AR system. To reduce the out-of-focus fluorescence background, samples were first illuminated with the imaging/deactivation laser at a low incidence angle to deactivate fluorophores above and below the focal plane, then a highly oblique incidence geometry with incidence angle only slightly smaller than the critical angle was used for activation and excitation, restricting illumination to a 2–3 μm depth into the cell sample. Typically, one STORM image acquired in several minutes covers an imaging volume of 81.92 μm×81.92 μm×750 nm without the need of sample scanning.

For STORM experiments, all cells were mounted in imaging buffer made by mixing the following four solutions with a volume ratio of 80:10:10:1 immediately before applying to the cells: DPBS, 1 M mercaptoethylamine with pH adjusted to 8.5 using HCl, 50% glucose solution in water, and an antibeaching oxygen scavenger system (10 mg of glucose oxidase+25 ml of catalase and 100 ml of DPBS, mix well and centrifuge for 1 min). Clean coverglass was placed on top of the sections and excess imaging buffer was removed followed by sealing the edges with nail polish.

#### Bead imaging

We used the plasmonic emission from 80 to 100 nm gold (Au) nanoparticles sparsely adsorbed (∼2000 per mm^2^) to the coverglass surface and immobilized by 30–50 nm of sputtered SiO_2_. Fiducialed coverglasses were ultraviolet-sterilized (15 min), rinsed with DPBS before imaging. During imaging of fiducial beads, 100 mW 647 laser was used to acquire sample points at different *z* positions.

#### 2D Gaussian fit of STORM images

Due to insertion of cylindrical lens, the signal of individual point was elongated along *x* axis or *y* axis depending on the position of the point, above or below the focal plane, respectively. Therefore, during 2D Gaussian fit, the *x* and *y* directions were fitted separately. Combine the two fitting, position of the point could be extracted by identifying the *x* and *y* peaks.

#### Characterization of the photoswitching properties of Alexa Fluor 647

AlexaFluor 647 donkey anti-mouse antibody (Life Technologies, Carlsbad, CA) was diluted 1:2000 in DPBS, sonicated for 15 min, and incubated with a clean coverslip. The sample was imaged under standard STORM imaging condition. The diluted antibody molecules were sparsely and non-specifically adhered to the coverslip, allowing single molecule events corresponding to each individual antibody molecule to be identified.

#### Characterization of *z* positions

As described in previous section, 2D Gaussian fits have the X-Y ellipticity that depends on the vertical position of the sample. We used the following definition for X-Y ellipticity:
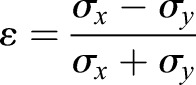
where *σ_x_* and *σ_y_* are the standard deviations (Gaussian widths) of the corresponding Gaussian fits. We measured the dependence of X-Y ellipticity *ε* on the vertical position of the point source for a field of ∼40 Au nanoparticles, using a polynomial fit between ellipticity and *z* positions. Thus, each point from STORM imaging was correlated with the fitting to extract the *z* positions.

#### Quantification of E-cadherin cluster size and density

Mean shift clustering is based on the multivariate kernel density function, which indicates the point density in certain dimensions:
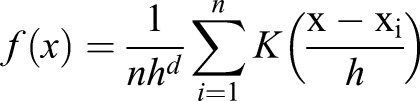
where n is the number of data points, d is the dimension, in this case, d=2, K is the profile of the kernel, which integrates to one, and h defines the radius of kernel. Taking the gradient of the density estimator gives the direction pointing toward the maximum increase in density. After several iterations from the randomly picked points, the local maxima can be obtained as the cluster centers. In this work, Matlab codes were developed for Mean-shift algorism. After all clusters were identified, the cluster size and density were characterized by diameter of cluster and points in one cluster, respectively.
